# Butanol as a major product during ethanol and acetate chain elongation

**DOI:** 10.3389/fbioe.2023.1181983

**Published:** 2023-05-18

**Authors:** Aide Robles, Skanda Vishnu Sundar, Srivatsan Mohana Rangan, Anca G. Delgado

**Affiliations:** ^1^ Biodesign Swette Center for Environmental Biotechnology, Arizona State University, Tempe, AZ, United States; ^2^ School of Sustainable Engineering and the Built Environment, Arizona State University, Tempe, AZ, United States; ^3^ Engineering Research Center for Bio-Mediated and Bio-Inspired Geotechnics, Arizona State University, Tempe, AZ, United States

**Keywords:** butanol, butyrate, hydrogen partial pressure, carboxylate reduction, *Clostridium kluyveri*, microbial chain elongation, total gas pressure

## Abstract

Chain elongation is a relevant bioprocess in support of a circular economy as it can use a variety of organic feedstocks for production of valuable short and medium chain carboxylates, such as butyrate (C4), caproate (C6), and caprylate (C8). Alcohols, including the biofuel, butanol (C4), can also be generated in chain elongation but the bioreactor conditions that favor butanol production are mainly unknown. In this study we investigated production of butanol (and its precursor butyrate) during ethanol and acetate chain elongation. We used semi-batch bioreactors (0.16 L serum bottles) fed with a range of ethanol concentrations (100–800 mM C), a constant concentration of acetate (50 mM C), and an initial total gas pressure of ∼112 kPa. We showed that the butanol concentration was positively correlated with the ethanol concentration provided (up to 400 mM C ethanol) and to chain elongation activity, which produced H_2_ and further increased the total gas pressure. In bioreactors fed with 400 mM C ethanol and 50 mM C acetate, a concentration of 114.96 ± 9.26 mM C butanol (∼2.13 g L^−1^) was achieved after five semi-batch cycles at a total pressure of ∼170 kPa and H_2_ partial pressure of ∼67 kPa. Bioreactors with 400 mM C ethanol and 50 mM C acetate also yielded a butanol to butyrate molar ratio of 1:1. At the beginning of cycle 8, the total gas pressure was intentionally decreased to ∼112 kPa to test the dependency of butanol production on total pressure and H_2_ partial pressure. The reduction in total pressure decreased the molar ratio of butanol to butyrate to 1:2 and jolted H_2_ production out of an apparent stall. *Clostridium kluyveri* (previously shown to produce butyrate and butanol) and *Alistipes* (previously linked with butyrate production) were abundant amplicon sequence variants in the bioreactors during the experimental phases, suggesting the microbiome was resilient against changes in bioreactor conditions. The results from this study clearly demonstrate the potential of ethanol and acetate-based chain elongation to yield butanol as a major product. This study also supports the dependency of butanol production on limiting acetate and on high total gas and H_2_ partial pressures.

## 1 Introduction

Microbial production of renewable and carbon-neutral chemicals is an important avenue to reduce carbon output and support a circular economy. The carboxylate platform, a microbial-based approach with global traction, can use diverse organic streams as feedstocks for production of butyrate (C4), caproate (C6), and caprylate (C8) (Steinbusch et al., 2010; Agler et al., 2011; Spirito et al., 2014; Roghair et al., 2018; De Groof et al., 2019; Han et al., 2019). These carboxylates are specialty chemicals for applications such as animal feed supplements, additives in chemical manufacturing, and biofuel precursors (Chen et al., 2017; Han et al., 2019; Wang and Yin, 2022). Chain elongation, the central metabolic process in the carboxylate platform, uses the reverse β-oxidation pathway to increase the carbon chain lengths by two carbons per completed cycle (Spirito et al., 2014; Angenent et al., 2016). In ethanol and acetate-based chain elongation, bacteria couple the oxidation of ethanol to the reductive elongation of the carboxylate. *C. kluyveri*, a strictly chain-elongating and model organism, is one of multiple key players in reactor microbiomes for chain elongation (Barker and Taha, 1942; Seedorf et al., 2008; Angenent et al., 2016; Candry and Ganigué, 2021).

Alcohols, such as butanol (C4) and hexanol (C6), can also be generated in ethanol and acetate-based chain elongation (Steinbusch et al., 2010; de Leeuw et al., 2021; Joshi et al., 2021; Robles et al., 2021; Huo et al., 2022), but their production is not always measured or reported. The value of an alcohol typically increases with carbon chain length (e.g., butanol vs. hexanol) because the higher carbon content yields the molecule a higher energy content and stability (Schiel-Bengelsdorf et al., 2013; Fernández-Naveira et al., 2017). Two pathways have been proposed for butanol production during chain elongation: (1) hydrogenotrophic carboxylate reduction (e.g., butyrate reduction to butanol) (Steinbusch et al., 2008), and (2) carboxyl-hydroxyl exchange, which couples excessive (hydrogenogenic) ethanol oxidation with hydrogenotrophic carboxylate reduction (de Leeuw et al., 2021). Butanol concentrations produced in chain elongation studies have ranged from trace concentrations up to 227 mM C (Richter et al., 2016). Several studies have reported concentrations on the range of 15–60 mM C butanol under a variety of chain elongation conditions in batch, semi-batch, or continuous bioreactors (Steinbusch et al., 2008; Ganigué et al., 2016; de Leeuw et al., 2021; Joshi et al., 2021; Robles et al., 2021; Fernández-Blanco et al., 2022; Huo et al., 2022; Vees et al., 2022; Bäumler et al., 2023). However, the conditions under which chain-elongating microbiomes can be directed to yield butanol as a major product during ethanol and acetate chain elongation are unknown.

Optimizing bioreactor conditions, identifying key microorganisms, and broadening the spectrum of possible end-products, including butanol and longer alcohols, are research endeavors required to increase the relevance of chain elongation as a bioprocess for a circular economy (de Leeuw et al., 2019; Han et al., 2019; Wang and Yin, 2022; Shrestha et al., 2023). Butanol can be used directly as a fuel or mixed with gasoline (Dekishima et al., 2011). It is also used as a sustainable solvent, a chemical intermediate, and it is used in the production of common-use items, such as cosmetics and pharmaceuticals (Ndaba et al., 2015). Butanol may also be a desirable H_2_-releasing substrate in subsurface bioremediation applications under anoxic conditions. For example, reductive dehalogenation of chlorinated ethenes (i.e., tetrachloroethene, trichloroethene, and vinyl chloride) by *Dehalococcoides mccartyi* sp. requires H_2_ as the obligate electron donor to convert the contaminants to non-toxic ethene (Löffler et al., 2013; Delgado et al., 2014; Mohana Rangan et al., 2020). Butanol has been shown to promote the complete reductive dehalogenation of tetrachloroethene and to enhance dehalogenation rates when tetrachloroethene was present as a dense non-aqueous phase liquid (Yu and Semprini, 2009). More recently, ethanol and acetate chain elongation was shown to support reductive dehalogenation of trichloroethene directly through production of H_2_ during elongation of acetate and indirectly through fermentation of the chain elongation products, butyrate and butanol (Robles et al., 2021). Understanding the conditions under which chain elongation may be steered to produce butanol and its precursor, butyrate, benefits applications for biochemical production and bioremediation.

The most studied pathway for bio-butanol production is acetone-butanol-ethanol (ABE) fermentation. In ABE fermentation, sugars and starches are converted to carboxylates (acetate and butyrate) and solvents (acetone, butanol, and ethanol) (Ndaba et al., 2015). Concentrations on the order of 539–1,000 mM C butanol (10–20 g L^−1^) (Xu et al., 2015; Wechgama et al., 2017) have been achieved *via* ABE fermentation with butanol yields ranging from ∼0.2 to 0.4 g g^−1^ glucose (Ndaba et al., 2015; Veza, Muhamad Said and Latiff, 2021). Butanol production *via* ABE is influenced by the medium composition (Al-Shorgani et al., 2018b), carbon source (Al-Shorgani et al., 2012), temperature (Ramió-Pujol et al., 2015), concentration of butyrate (Lee et al., 2008), and pH (Bahl et al., 1982), among other parameters. A limited number of chain elongation studies have reported that production of butanol by chain-elongating microbiomes can be encouraged by combining butyrate with a high H_2_ partial pressure (around 150 kPa) (Steinbusch et al., 2008) and by feeding syngas in bioreactors at an initial pH of around 6 (Ganigué et al., 2016). In anaerobic systems, including ABE fermentation, the total gas pressure in a bioreactor has also been shown to influence metabolic shifts from acidogenesis to solventogenesis (Doremus et al., 1985; Brosseau et al., 1986; Yan et al., 2017). Nonetheless, the total gas pressure is rarely directly considered in bioreactor design for chain elongation.

In this study, we investigated production of butanol (and its precursor butyrate) during ethanol and acetate-based chain elongation in semi-batch bioreactors (0.16 L serum bottles) fed with a range of ethanol concentrations (100–800 mM C), a constant and limiting acetate concentration (50 mM C), and an initial partial pressure of ∼112 kPa. Under conditions of high total gas pressure and high H_2_ partial pressure, we found that increasing the concentration of ethanol was positively correlated to chain elongation activity and ultimately yielded a butanol:butyrate molar ratio of 1:1. A butanol concentration of 114.96 ± 9.26 mM C was achieved in this study. The dependency of butanol production on high total gas pressure and high H_2_ partial pressure was experimentally verified by releasing gas from bioreactors. This study is the first to show butanol as a major end-product of ethanol and acetate chain elongation.

## 2 Materials and methods

### 2.1 Medium composition and microbial inoculum

Anaerobic mineral medium was prepared as described in Robles et al. (2021). The medium contained 10 mL of a salt stock solution, 1 mL of a trace element A solution, 1 mL of a trace element B solution, and 1 mL of a vitamin solution per liter. The solutions were prepared as described in Löffler et al. (2005) with the modifications described in Robles et al. (2021). Additionally, the medium was amended with the reductants Na_2_S (0.2 mM) and L-cysteine (0.4 mM), vitamin B_12_ (0.5 mg L^−1^) and the buffer, potassium phosphate (10 mM). The initial pH of the medium was 7.5. The medium was bottled with UHP N_2_ in the headspace. Ethanol, 200 proof, molecular biology grade (Sigma Aldrich, St. Louis, MO, United States) and ReagentPlus sodium acetate trihydrate (Sigma Aldrich) were used as substrates for the bioreactors.

The inoculum for this study was an enrichment culture from a soil microcosm. The microcosm contained 10 g soil from Phoenix Goodyear Airport-North Superfund site. The soil microcosm was inoculated with 5 mL of a lactate-fermenting and trichloroethene-dehalogenating culture and 5 mL of an ethanol- and acetate-chain elongating culture in 90 mL anaerobic medium with 100 mM C ethanol, 100 mM C acetate, and 2.1 mmol L^−1^ trichloroethene (Robles et al., 2021). The enrichment culture from this soil microcosm converted ethanol and acetate to mainly butyrate with minimal methane production (≤1.7 mmol L^−1^ methane observed during incubation). The soil microcosm enrichment culture was maintained under these conditions prior to use in the experiments from this study. The microbial community composition of the enrichment culture primarily consisted of members of *Clostridiales*, *Burkholderiales*, and *Eubacteriales*. The most notable chain elongating microorganism in the culture was *Clostridium kluyveri* [100% similar to strain K1, ATCC 8527/DSM 555 using BLAST + consensus taxonomy classifier plugin (Camacho et al., 2009)].

### 2.2 Experimental setup

Experiments in triplicate were setup in serum bottles sealed with rubber stoppers and aluminium crimps. The total volume of the bottles was 0.16 L (160 mL) with an initial liquid volume of 75 mL. For brevity, we hereafter refer to the experimental serum bottle reactors as bioreactors. The bioreactors were provided with the following initial concentration of substrates: 100 mM C ethanol + 50 mM C acetate, 200 mM C ethanol + 50 mM C acetate, 400 mM C ethanol + 50 mM C acetate, 800 mM C ethanol + 50 mM C acetate, and 800 mM C ethanol (Supplementary Table S1). Each bioreactor received 6 mL of inoculum culture at the start of the experiment. The bioreactors were operated in semi-batch cycles with draw and fill performed every 7 days. At the end of each 7-d cycle, one-third of the bioreactor’s liquid contents (25 mL) was removed and replaced with fresh medium (25 mL) containing the same initial substrate concentration as in Supplementary Table S1. The initial total gas pressure in the bioreactors was set at 110 ± 2 kPa (∼1.08 atm) by injecting UHP N_2_ gas. The initial pH in the bioreactors was set to ∼7.5. During the first seven semi-batch cycles, the bioreactors were allowed to accumulate H_2_ in the headspace and thus increasing total gas pressure. A pseudo steady-state was achieved by semi-batch cycle 4. The operating phase in semi-batch cycles 4 through 7 is referred to in the text and figures as “High H_2_ & total pressure.” At the beginning of semi-batch cycle 8, the bioreactors were intentionally perturbed by decreasing the total gas pressure to ∼112 kPa (similar to time 0 conditions). The operating phase for semi-batch cycles 8 through 11 is referred to in text and figures as “High H_2_ & low total pressure.” A total of 11 semi-batch cycles were completed in the study. The condition labelled “100 mM C EtOH + Acetate” was resupplied with 240 mM C ethanol at the beginning of cycle 8. An abiotic control with 200 mM C ethanol and 50 mM C acetate was setup and operated for two cycles. All bioreactors were incubated at 31°C on a platform shaker set to 150 rpm.

### 2.3 Chemical analyses

Ethanol, butanol, and hexanol, and acetate, butyrate, caproate, and caprylate were quantified at the beginning and end of each semi-batch cycle, except cycles 6 and 10 where samples were not preserved for analysis. The concentrations of carboxylates and alcohols were determined using a high-performance liquid chromatograph (HPLC) equipped with a refractive index detector, a photodiode-array detector, and an Aminex HPX-87H column (Bio-Rad Laboratories, Hercules, CA, United States). The HPLC method and sample preparation were completed as previously described (Joshi et al., 2021; Miranda et al., 2022). The method was run for a total of 120 min, with retention times ranging from 15 min for acetate to 79 min for hexanol. The detection limit of the analytes was 0.02–0.05 mM. Hexanol and caprylate were not detected in samples from this study. pH measurements were taken using a benchtop pH meter (Orion 2-star, Thermo Scientific, Waltham, MA, United States) equipped with an economy series pH electrode.

H_2_ concentration in the headspace of the bioreactors was quantified using a gas chromatograph with a thermal conductivity detector (GC-TCD) (Shimadzu GC-2010, Columbia, MD, United States) and a fused silica capillary column (Carboxen 1010 PLOT column, Supelco, Bellefonte, PA, United States). The sampling and GC-TCD method details were as previously published (Robles et al., 2021; Meinel et al., 2022). The calibration range for H_2_ was 0.013–10.22 mmol L^−1^ gas. The total gas volume in the bioreactors was measured with a frictionless syringe (Sigma-Aldrich) and was converted to a total gas pressure as described in the Supplementary Material.

### 2.4 DNA extraction and microbial community analysis

DNA was extracted from bioreactor samples preserved in RNAprotect cell reagent (Qiagen, Germantown, MD, United States) at −80°C at the end of semi-batch cycles 5 (during “High H_2_ & total pressure” phase) when butanol concentrations were highest and 9 (during “High H_2_ & low total pressure” phase) when butanol concentrations were lowest. Pellets were pre-treated with an enzyme lysis buffer containing 20 mM Tris·HCl, 2 mM EDTA, 250 μg mL^−1^ achromopeptidase, and 20 mg mL^−1^ lysozyme (Mohana Rangan et al., 2023). After pre-treatment, genomic DNA was extracted using the Qiagen DNeasy Blood and Tissue kit (MO BIO Laboratories Inc., Carlsbad, CA, United States) following the protocol for Gram-positive bacteria.

Microbial community amplicon sequencing was performed on the Illumina platform with a Miseq instrument (San Diego, CA, United States) at the ASU Genomics Core Facility, Arizona State University, Tempe, AZ, United States. Sequencing used the universal primers 515F and 806R for the V4 hyper-variable region of the 16S rRNA gene of *Bacteria* and *Archaea* (Caporaso et al., 2012). Forward and reverse reads were processed using the Quantitative Insights into Microbial Ecology (QIIME 2.0 v. 2022.2) pipeline (Bolyen et al., 2019). Each sequence was truncated at 232 base pairs using DADA2 to maintain a quality score of 25 or better and produce amplicon sequence variants (ASVs). A pretrained Naïve Bayes classifier referencing the SILVA database (v.138) (Quast et al., 2012; Bokulich et al., 2018; Robeson et al., 2021) and the q2-feature-classifier plugin were used to assign taxonomy to amplicon sequence variants (DeSantis et al., 2006). For alpha diversity, Pielou’s evenness index was determined from sequences rarefied to a sampling depth of 16,224 counts. The raw sequences were submitted to the NCBI Sequence Read Archive and are available under the project number PRJNA913573 with accession numbers SRX18767348–SRX18767367.

The 16S rRNA gene of *C. kluyveri* was quantified in bioreactors at the end of semi-batch cycles 5 (“High H_2_ & total pressure”) and 9 (“High H_2_ & low total pressure”) *via* quantitative real-time PCR (qPCR) (Bio-Rad CFX96). The qPCR assays contained the following per 25 µL reaction: 2 µL DNA, 1.125 μL F′ primer, 1.125 μL R′ primer, 12.5 µL SYBR Green Master Mix (Bio-Rad) and 8.25 µL RNase-free water (MO Bio Laboratories Inc.). No-template controls were also included in the qPCR runs. A six-point calibration curve was created using a gBlock fragment (Integrated DNA Technologies, Inc., Coralville, IA, United States) as shown in the Supplementary Material. Triplicate reactions were setup for experimental samples, no-template controls, and the calibration. To infer concentrations of *C. kluyveri* in the bioreactors (in cells mL^−1^), copies per mL of the 16S rRNA gene were divided by 7, which is the number of 16S rRNA gene copies in the chromosomal DNA of *C. kluyveri* (Stoddard et al., 2014). Additional details about the qPCR analysis are presented in the Supplementary Material.

### 2.5 Calculations and statistical analysis

The concentrations of carbon (C)-containing chain elongation substrates and products were converted to mM carbon (C) by multiplying the concentration in mM by the corresponding C atom number in each compound: ethanol, 2; acetate, 2; butanol, 4; butyrate, 4; and caproate, 6. Total gas pressure was determined using the measured total gas volume and the ideal gas law. The average and maximum rates of butyrate and butanol production in units of mmol C L^−1^ d^−1^ were calculated using the data from the “High H_2_ & total pressure” phase (semi-batch cycles 4–7). Average ratios of butanol to butyrate (mol:mol) produced were calculated using data from semi-batch cycles 4–7 (for “High H_2_ & total pressure” phase) and 11 (three cycles after pressure was reduced to ∼112 kPa from “High H_2_ & low total pressure” phase). A Student’s *t*-test was used to evaluate statistical significance of chemical and microbiological data with a 95% confidence interval (*p* < 0.05 was considered statistically significant). Additional details on calculations are in the Supplementary Material.

## 3 Results and discussion

### 3.1 Bioreactors with 400 mM C ethanol and 50 mM C acetate achieve a 1:1 butanol to butyrate molar ratio during “High H_2_ & total pressure” phase

**FIGURE 1:**
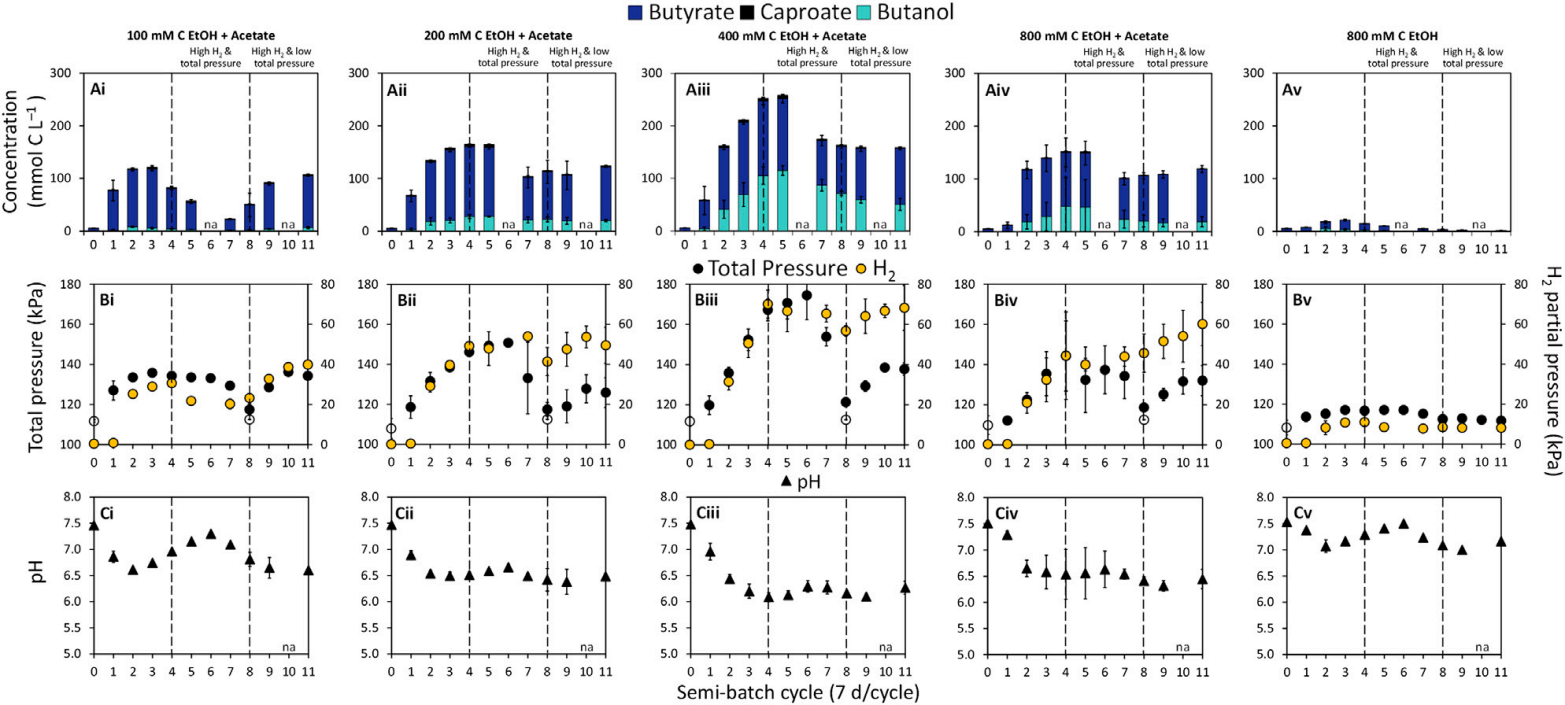
Concentrations of **(A)** butyrate, caproate, and butanol, **(B)** total gas pressure and H_2_ partial pressure (yellow circles on secondary y-axis), and **(C)** pH in semi-batch bioreactors. The concentration of acetate in the bioreactors was 50 mM C. The empty symbols for total pressure are measurements at time 0 and beginning of cycle 8 when total pressure was decreased to ∼112 kPa. The filled symbols are measurements at the end of a semi-batch cycle. The data are averages with standard deviations of triplicate bioreactors. The pH at the start of each cycle is reported in Supplementary Table S1. na = not analyzed.

Semi-batch bioreactors probing the potential of chain elongation for butanol production were fed with ethanol (100–800 mM C) and acetate (50 mM C) or ethanol only (800 mM C) at an initial total gas pressure of ∼112 kPa. Consumption of substrates (Supplementary Figure S1) coupled to the production of butyrate and butanol was observed in all bioreactors (Figures 1Ai–Av). In the absence of the inoculum culture, substrate consumption was not observed (Supplementary Figure S2). Increasing the ethanol concentration from 100 mM C to 400 mM C while keeping the acetate concentration constant enhanced chain elongation activity, including butanol production (Figures 1Ai–Aiii). It has been previously documented that a higher ethanol concentration and/or a higher molar ratio of ethanol to acetate can steer chain elongation from mainly butyrate to caproate and/or caprylate (Steinbusch et al., 2010; Coma et al., 2016; Liu et al., 2016; Lonkar et al., 2016; Spirito et al., 2018; Joshi et al., 2021). In our previous work, we showed that soil slurry semi-batch bioreactors fed with 200 mM C ethanol and 200 mM C acetate produced between 11.41 and 59.89 mM C butanol and 1.10 and 31.77 mM C hexanol (Joshi et al., 2021). However, butyrate, caproate, and caprylate were the dominant products (Joshi et al., 2021). In the present study, no caprylate or hexanol was detected and the caproate concentration remained low in all bioreactors throughout operation (0.37 ± 0.01–6.48 ± 0.87 mM C caproate, Figures 1Ai–Av). Two connected reasons likely explain the limited production of caproate. First, acetate, the electron acceptor in chain elongation, was limiting in our bioreactors relative to the concentration of ethanol, the electron donor. Partial ethanol oxidation to acetate and H_2_ is the ATP yielding reaction in chain elongation (Seedorf et al., 2008). Ethanol oxidation would provide a required carboxylate electron acceptor for chain elongation. However, the high H_2_ partial pressure in our bioreactors made this reaction unfavorable after four semi-batch cycles as evident by the plateau in H_2_ concentration (Figures 1Bii–Biv). Thus, butanol production became a major pathway through which electrons from H_2_ could be consumed in the system.

Butanol production was observed at H_2_ partial pressures above ∼8.16 kPa, while significant butanol production occurred at H_2_ partial pressures above ∼50 kPa (*p* = 0.0241, Figures 1Bi–Bv). The highest concentration of butanol achieved in this study was 114.96 ± 9.26 mM C (∼2.13 g L^−1^) in condition “400 mM C EtOH + Acetate” at the end of semi-batch cycle 5 during the “High H_2_ & total pressure” phase (Figure 1Aiii). To the best of our knowledge, this is the highest reported butanol concentration in an ethanol and acetate-based chain elongation study. At the end of semi-batch cycle 5, the bioreactors fed with 400 mM C ethanol reached a H_2_ partial pressure of 66.58 ± 3.99 kPa and a total gas pressure of 170.63 ± 14.35 kPa (Figure 1Biii). Steinbusch et al. (2008) documented that bioreactors with a H_2_ headspace (150 kPa H_2_) and 200 mM C butyrate produced up to 14.64 mM C butanol. Additionally, total gas pressures between 156 and 184 kPa previously showed selection for alcohols over carboxylates in methanogenic bioreactors containing H_2_ and CO_2_ in the headspace (Yan et al., 2017). Findings from our study suggest that H_2_ partial pressure in combination with total gas pressure are powerful selection tools for butanol.

The highest butanol concentrations were achieved under conditions where H_2_ production stalled during the “High H_2_ & total pressure” phase (Figure 1, cycle 5). Enhanced production of butanol under this H_2_ stall phenomenon has been observed in ABE fermentation (Doremus et al., 1985; Brosseau et al., 1986; Yan et al., 2017). Stalls in H_2_ production can be attributed to the influence of dissolved H_2_ on NADH_2_ and H_2_ supersaturation in the medium, which can inhibit H_2_ production *via* the ferredoxin-linked hydrogenase (Doremus et al., 1985). Under this scenario, microorganisms can channel electrons through NADH:ferredoxin oxidoreductase, reducing butyrate to butanol (Doremus et al., 1985), supporting the high butanol production observed in our study.

Butanol (and butyrate) production rates in bioreactors increased with increasing concentration of ethanol up to 400 mM C (Supplementary Figure S3). The optimal range for ethanol-based chain elongation using mixed cultures has been reported to be between 216 and 434 mM C ethanol (5–10 g L^−1^ ethanol) (Lonkar et al., 2016). Improved production rates from our bioreactors with 200 and 400 mM C ethanol (+acetate) are in line with these previous findings. The bioreactors also showed a clear trend with respect to the molar ratio of butanol to butyrate produced. Specifically, the butanol:butyrate molar ratio increased from ∼1:6 at 100 mM C ethanol (+acetate) to ∼1:1 at 400 mM C ethanol (+acetate) during the “High H_2_ & total pressure” phase (Table 1).

**TABLE 1:** Average butanol to butyrate molar ratio in bioreactors during semi-batch cycles 4–7 (“High H_2_ & total pressure” phase) and cycle 11 (“High H_2_ & low total pressure” phase).

Bioreactor label/condition	Butanol:butyrate (mol:mol) during experimental phases	
	High H_2_ & total pressure	High H_2_ & low total pressure
100 mM C EtOH + Acetate	1:6	1:9
200 mM C EtOH + Acetate	1:3	1:4
400 mM C EtOH + Acetate	1:1	1:2
800 mM C EtOH + Acetate	1:3	1:6

Chain elongation activity in bioreactors significantly decreased when the concentration of ethanol was increased from 400 to 800 mM C (+50 mM C acetate) (*p* = 0.0002, Figure 1Aiii; Supplementary Figure S4). The decrease in chain elongation activity in bottles fed 800 mM C ethanol and 50 mM C acetate was likely a consequence of ethanol inhibition. Concentrations between ∼600 and 1,720 mM C ethanol (14 and 40 g L^−1^ ethanol) have been previously reported as inhibitory in chain elongation studies (Kucek et al., 2016; Lonkar et al., 2016). Butanol concentration of ∼50 mM C (1 g L^−1^) have been reported to inhibit growth of the butanol producer *C. carboxidivorans* (Fernández-Naveira et al., 2016). In our mixed culture bioreactors, ∼100 mM C butanol did not appear to have a prominent inhibitory effect as approximately the same percentage of substrates went to production of butyrate, butanol, caproate and H_2_ in bioreactors with 200 mM C ethanol and 400 mM C ethanol (Supplementary Figure S4). The absence of added electron acceptor, acetate, in the “800 mM C EtOH” condition further suppressed chain elongation activity, in agreement with previous studies (Spirito et al., 2018; de Leeuw et al., 2021; Joshi et al., 2021). Thus, the poor chain elongation extent at 800 mM C ethanol (Figure 1Av) is reflective of ethanol inhibition and acetate limitation.

A general decrease in pH was observed in all ethanol and acetate bioreactors from the start of incubation (time 0) and by the end of each semi-batch cycle (Figures 1Ci–Cv, pH at the start of cycles in Supplementary Table S2). The pH range across ethanol and acetate conditions was 6.09 ± 0.09–7.30 ± 0.04 at the end of semi-batch cycles (Figures 1Ci–Civ). The lowest pH was observed in “400 mM C EtOH + Acetate” bioreactors, where the highest butanol production occurred (pH ranged from 6.09 ± 0.09 to 6.29 ± 0.11, Figure 1Ciii). In ABE fermentation, butyrate in combination with mildly acidic pH (4.5–6.2) is a trigger for butanol production and increases solvent yields (Matta-El-Ammouri et al., 1987; Tashiro et al., 2004; Li et al., 2011; Al-Shorgani et al., 2018a). A pH range between 4.7 and 6.4 has also been found to support alcohol production in chain elongation systems with *C. kluyveri* (Ganigué et al., 2016; Richter et al., 2016). In this study, higher butanol production was observed at the pH range 6.1–6.3; however, the pH was not controlled during the experiment and thus a relationship between the extent of butanol production and pH could not be discerned based on the experimental design.

### 3.2 Perturbations in the total gas pressure confirm the dependency of butanol production on gas pressure and composition

To evaluate the dependency of butanol production on gas pressure and composition, the total gas pressure was adjusted at the start of semi-batch cycle 8 by releasing gas from the bioreactors and resetting the total gas pressure to ∼112 kPa (Figure 1, “High H_2_ & low total pressure” phase). The decreases in total gas pressure immediately decreased butanol production at the end of cycle 8 (Figures 1Aii–Aiv) but butanol production continued throughout this experimental phase. The H_2_ stall observed during the first phase was overcome during the “High H_2_ & low total pressure” phase. During the second phase, H_2_ partial pressures reached 7.75 ± 1.33–68.19 ± 1.33 kPa and total gas pressure ranged from 111.71 ± 1.33 to 138.49 ± 1.33 kPa (Figures 1Bi–Bv). The butanol:butyrate molar ratio was consistently lower in all conditions during the “High H_2_ & low total pressure” phase after the total gas pressure was decreased (Figures 1Ai–Aiv; Table 1). The lower total gas pressure observed in “High H_2_ & low total pressure” phase and lower butanol:butyrate molar ratios supports previous findings where total gas pressures between 101 and 124 kPa previously selected for production of carboxylate and H_2_ over alcohols (Yan et al., 2017).

### 3.3 Butyrate and butanol producing microbial community showed stability and resilience during changes in bioreactor conditions

The microbial community composition was determined to identify any potential linkage between observed activity, particularly butanol and butyrate production, during the “High H_2_ & total pressure” phase and the “High H_2_ & low total pressure” phase. Regardless of the experimental phase, the most abundant or second most abundant phylum in the microbial community was *Firmicutes* (recently renamed *Bacillota*) (Supplementary Figure S5). *Firmicutes* contains the majority of the identified chain elongating species (Elsden et al., 1956; Wallace et al., 2004; Seedorf et al., 2008; Angenent et al., 2016; Zhu et al., 2017; Han et al., 2018; Candry and Ganigué, 2021; Joshi et al., 2021). During the “High H_2_ & total pressure” phase, *Firmicutes* ASVs accounted for 76.2% of the microbial community in bioreactors fed 400 mM C ethanol and acetate, which also produced the highest concentration of butanol and butyrate (Figure 1Aiii; Supplementary Figure S5). ASVs for strict carboxydotrophic microbes known to produce butanol were not identified in these bioreactors. Furthermore, methanogenic ASVs were either not detected in samples or were at ≤ 0.7% of total sequences, consistent with the characteristics of the inoculum where methane production was absent or minimal even at a pH range of 6–7 (Joshi et al., 2021; Robles et al., 2021).

**FIGURE 2:**
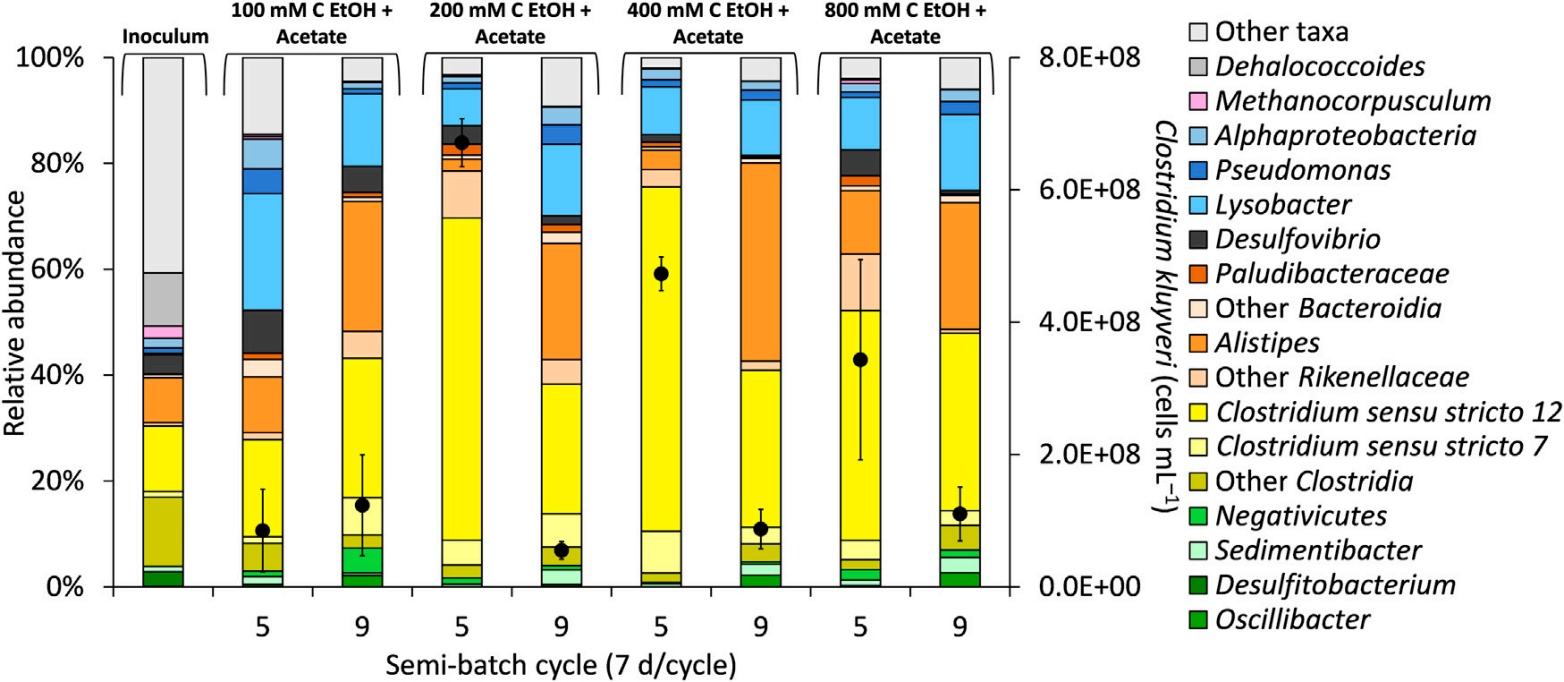
Selected taxa in the bioreactors and quantification of *Clostridium kluyveri* (black circles, secondary y-axis) during “High H_2_ & total pressure” phase (end of semi-batch cycle 5) and during “High H_2_ & low total pressure” phase (end of semi-batch cycle 9). The concentration of acetate in the bioreactors was 50 mM C. The total pressure in the bioreactors was decreased at the end of semi-batch cycle 7. The data are averages from triplicate bioreactors.

In our study, *C. kluyveri* ASVs, which classified in *Clostridium sensu stricto 12*, were most abundant in bioreactors fed 200 and 400 mM C ethanol (+acetate) in semi-batch cycle 5, accounting for ∼56% of the total sequences (Figure 2). In our study, the *C. kluyveri* ASV showed 100% sequence match to strain K1 (Barker and Taha, 1942) in a BLAST consensus sequence search. *C. kluyveri* produces carboxylates as the major metabolites. Butanol (Thauer et al., 1968; Kenealy and Waselefsky, 1985) and propanol (Candry et al., 2020) have also been reported in pure culture studies with *C. kluyveri*. It is possible that *C. kluyveri* was a major player in butanol production, although other microorganisms could have contributed to production of this metabolite. Quantification of *C. kluyveri* through qPCR supported its prominent role in chain elongation (and potentially butanol production). Specifically, the highest concentration of *C. kluyveri* cells (up to 6.71×10^8^ ± 3.61×10^7^ cells mL^−1^) was observed during the “High H_2_ & total pressure” phase at 200 mM C ethanol (Figure 2, cycle 5). A lower concentrations of *C. kluyveri* was quantified at 800 mM C ethanol than at 200 mM C ethanol (+acetate), even though the cumulative concentration of butyrate and butanol were similar in these conditions (Figures 1Aii, Aiv).

Microbial diversity and richness were not affected by increasing concentration of ethanol or by the experimental phase (Supplementary Figure S6, Pielou’s evenness range 0.49–0.67, *p* = 0.4199), highlighting the overall stability of the microbiome. A noteworthy trend was observed during “High H_2_ & low total pressure” where *Clostridium sensu stricto 12* ASV decreased in relative abundance while *Alistipes* ASV (up to 37% of the total sequences, Figure 2) and other *Rikenellaceae* ASVs from *Bacteroidota* increased in relative abundance (Figure 2). *Alistipes* has been recently linked to butyrate production in anaerobic digestion of cellulose using anaerobic sludge as inoculum (Rico et al., 2021). *Alistipes* ASVs possibly also contributed to butyrate production in our bioreactors. *Sedimentibacter*, *Oscillibacter*, and *Pseudomonas* are ASVs commonly reported to also enrich in chain elongation bioreactors (Rühl et al., 2009; Candry and Ganigué, 2021; Joshi et al., 2021). In our study, these ASVs were more abundant during the “High H_2_ & low total pressure” phase, although to a much lower extent than *Clostridium sensu stricto 12* and *Alistipes* (Figure 2).

## 4 Conclusion

In this study, we show that selective conditions in bioreactor can be imposed for a chain-elongating microbiome to yield butanol as a major product in ethanol and acetate-based chain elongation. The highest chain elongation activity was observed in bioreactors fed 400 mM C ethanol during the “High H_2_ & total pressure” phase where up to 114.95 ± 9.26 mM C butanol (∼2.13 g L^−1^) was produced. We showed that bioreactors operated under high total pressure and high H_2_ partial pressure with limited acetate relative to ethanol produce butanol instead of the longer carboxylate, caproate. The individual effect of H_2_ partial pressure, total gas pressure, and acetate concentration were not isolated in our work. However, results from this work clearly support that these parameters can be applied for selective production of butanol in chain elongation.

## Data Availability

The datasets presented in this study can be found in online repositories. The names of the repository/repositories and accession number(s) can be found below: https://www.ncbi.nlm.nih.gov/, PRJNA913573: SRX18767348–SRX18767367.
