# Supplementary material for: Butanol as a major product during ethanol and acetate chain elongation
Source: Front Bioeng Biotechnol. 2023 May 18;11:1181983. doi: 10.3389/fbioe.2023.1181983 (PMC10233103; doi:10.3389/fbioe.2023.1181983)
Supplement: Supplementary file 1 [file DataSheet1.docx]

Supplementary Material

Butanol as a Major Product during Ethanol and Acetate Chain Elongation

**Aide Robles^1, 2, 3,*^, Skanda Vishnu Sundar^1, 2^, Srivatsan Mohana Rangan^1, 2, 3^, and Anca G. Delgado^1, 2, 3, *^**

^1^Biodesign Swette Center for Environmental Biotechnology, Arizona State University, Tempe, AZ, USA

^2^School of Sustainable Engineering and the Built Environment, Arizona State University, Tempe, AZ, USA

^3^Engineering Research Center for Bio-mediated and Bio-inspired Geotechnics, Arizona State University, Tempe, AZ, USA

*** Correspondence:**Corresponding Authors
Anca G. Delgado: [anca.delgado@asu.edu](mailto:anca.delgado@asu.edu), Aide Robles: [arobles9@asu.edu](mailto:arobles9@asu.edu)

# Supplementary Data

**Total gas pressure and H_2_ calculations**

Total gas pressure ($P_{T}$, in units of kPa) was determined using the ideal gas law with Equation S1:

$P_{T}=\frac{P_{standard} \times V_{standard}}{V_{T}}$ (S1)

where P_standard_ is pressure at standard atmospheric pressure (101 kPa), V_standard_ is the volume of available headspace in the bioreactor (0.088L), and V_T_ is the total gas volume (L) (available headspace (0.088 L) plus the extra volume (L) obtained from readings using frictionless syringe). H_2_ data presented as partial pressure ($P_{H_{2}}$, in units of kPa) were calculated from gas concentrations (C_g_, in units of mmol L^−1^ gas) measured from gas samples of the bioreactors *via* gas chromatography using Equation S2:

$P_{H_{2}}=\frac{n_{H_{2}}\times R \times T}{V_{standard}}$ (S2)

where $n_{H_{2}}$ is the number of mol of H_2_, R is the gas constant (L kPa K^−1^ mol^−1^), and T is the incubation temperature (304.15 K).

**gBlocks design, qPCR standard calculations, and quantification of *Clostridium kluyveri***

A gBlocks gene fragment (Integrated DNA Technologies, Inc., Coralville, IA, USA) with 258 base pairs was designed using the 16S rRNA sequence of *C. kluyveri* ATCC 8527/DSM 555. The gene fragment design was as follows:

AggactactcgattcgtcgaacgtcGAGAAGCAATACCGCGAGGAGGAGCAAATCTCAAAAACTGCCCCCAGTTCGGATTGCAGGCTGAAACCCGCCTGCATGAAGTTGGAGTTGCTAGTAATCGCGAATCAGCATGTCGCGGTGAATACGTTCCCGGGTCTTGTACACACCGCCCGTCACACCATGAGAGCTGGCAACACCCGAAGTCCGTAGTCTAACCAAGGAGGACGCGAtcaagttagagcggtccacccgtt

where, the grey regions are generic adaptors to overcome high G/C content, the yellow region is the forward primer, and the green region is the reverse primer. The gBlock was received normalized to 500 ng (3138 fmol, dried) and was suspended in 50 µL RNAase-free water (MO BIO Laboratories Inc. Carlsbad, CA, USA) to reach a final concentration of 10 ng µL^−1^ (62.76 fmol µL^−1^). The concentration of the standard (C_std_, in units of ng µL^−1^) was verified using a Qubit with a dsDNA HS assay kit (Thermo Fisher Scientific, Waltham, MA, USA). The standard concentration as copies of the 16S rRNA gene of *C. kluyveri* (C_16S rRNA_, in units of 16S rRNA gene µL^−1^) was determined from the standard concentration (C_std_, in units of fmol µL^−1^) using Equation S3:

$C_{16S rRNA gene}=C_{std}\times\frac{1 mol}{1.0 \times{10}^{15} fmol} \times(6.022\times{10}^{23} {mol}^{-1})$ (S3)

where $6.022\times{10}^{23} {mol}^{-1}$ is Avogadro’s constant. The standard was then diluted to obtain the following calibration curve: 10^−1^, 10^−2^, 10^−3^, 10^−3^, 10^−4^, 10^−5^, and 10^−6^. To quantify concentrations of *C. kluyveri* cells mL^–1^, the concentrations in copies of the 16S rRNA gene µL^−1^ were converted to units per mL and divided by 7 (the number of 16S rRNA copies per *C. kluyveri* genome (Stoddard et al. 2014)).

The qPCR temperature conditions were adapted from previous publications (Weimer and Stevenson, 2012; Stevenson and Weimer, 2007): hold at 95°C for 10 min, 40 cycles of 95°C for 25 s and 59°C for 90 s, and a melting curve at 95°C for 15 s followed by 60 °C for 1 min and 95 °C for 1 s.

**Production rates, molar ratio, and distribution of electrons calculations**

The rates of butyrate and butanol production (∆C ∆t^−1^, in units of mmol C L^−1^ d^−1^) were calculated according to Equation S4:

$\frac{\Delta C}{\Delta t} =\frac{\left( C_{t2} \right)-(C_{t1})}{t_{2}-t_{1}}$ (S4)

where C_t_ is the concentration of the compound and t is the sampling time in days. Aqueous concentrations (mM) were converted to mM C by multiplying concentrations with the number of C atoms in each compound: butyrate, 4; butanol, 4; and caproate, 6. Molar ratios of butanol to butyrate produced (butanol:butyrate, in units of mol:mol) were calculated using Equation S5:

$butanol:butyrate=\frac{C_{butanol,t2}-C_{butanol,t1}}{C_{butanol,t2}-C_{butanol,t1}}:\frac{C_{butyrate,t2}-C_{butyrate,t1}}{C_{butanol,t2}-C_{butanol,t1}}$ (S5)

where C_butyrate,t2_ is the concentration of the compound at the end of a cycle and C_butyrate,t1_ is the concentration of the compound at the beginning of a cycle. Distribution of electrons from acetate and ethanol to end-products was calculated at semi-batch cycle 4 (beginning of “High H_2_ + gas pressure” phase) for all bioreactor conditions. The concentrations of substrates and end-products were converted to millielectron equivalents. The millielectron equivalents per mol values used in the calculations were as follows: H_2_, 2; acetate, 8; ethanol, 12; butyrate, 20; butanol, 26; and caproate, 32.

# Supplementary Tables

**Supplementary Table S1.** Experimental conditions setup in semi-batch bioreactors and operated for 11 cycles.

| Bioreactor label/condition | Total C from substrates (mM) | Ethanol  (mM) | Acetate  (mM) |
| --- | --- | --- | --- |
| 100 mM C EtOH + Acetate | 150 | 50 | 25 |
| 200 mM C EtOH + Acetate | 250 | 100 | 25 |
| 400 mM C EtOH + Acetate | 450 | 200 | 25 |
| 800 mM C EtOH + Acetate | 850 | 400 | 25 |
| 800 mM C EtOH | 800 | 400 | 0 |

**Supplementary Table S2**. pH in bioreactors *at the start of each semi-batch cycle*. The data are averages with standard deviation of triplicate bioreactors. na = not analyzed.

| Semi-batch cycle | Bioreactor label/condition | | | | |
| --- | --- | --- | --- | --- | --- |
|  | 100 mM C EtOH + Acetate | 200 mM C EtOH + Acetate | 400 mM C EtOH + Acetate | 800 mM C EtOH + Acetate | 800 mM C EtOH |
| 1 | 7.46 ± 0.02 | 7.49 ± 0.02 | 7.48 ± 0.01 | 7.51 ± 0.01 | 7.53 ± 0.01 |
| 2 | na | na | na | na | na |
| 3 | 6.87 ± 0.01 | 6.81 ± 0.04 | 6.76 ± 0.06 | 6.92 ± 0.10 | 7.23 ± 0.09 |
| 4 | 6.97 ± 0.02 | 6.81 ± 0.05 | 6.62 ± 0.09 | 6.89 ± 0.20 | 7.30 ± 0.03 |
| 5 | 7.15 ± 0.01 | 6.83 ± 0.03 | 6.56 ± 0.07 | 6.88 ± 0.30 | 7.42 ± 0.02 |
| 6 | 7.29 ± 0.01 | 6.87 ± 0.03 | 6.61 ± 0.05 | 6.89 ± 0.31 | 7.51 ± 0.01 |
| 7 | 7.10 ± 0.01 | 6.70 ± 0.04 | 6.45 ± 0.07 | 6.71 ± 0.18 | 7.23 ± 0.01 |
| 8 | 7.00 ± 0.00 | 6.59 ± 0.03 | 6.45 ± 0.8 | 6.63 ± 0.06 | 7.10 ± 0.01 |
| 9 | 6.80 ± 0.09 | 6.55 ± 0.15 | 6.38 ± 0.02 | 6.55 ± 0.05 | 7.00 ± 0.01 |
| 11 | na | na | na | na | na |

# Supplementary Figures


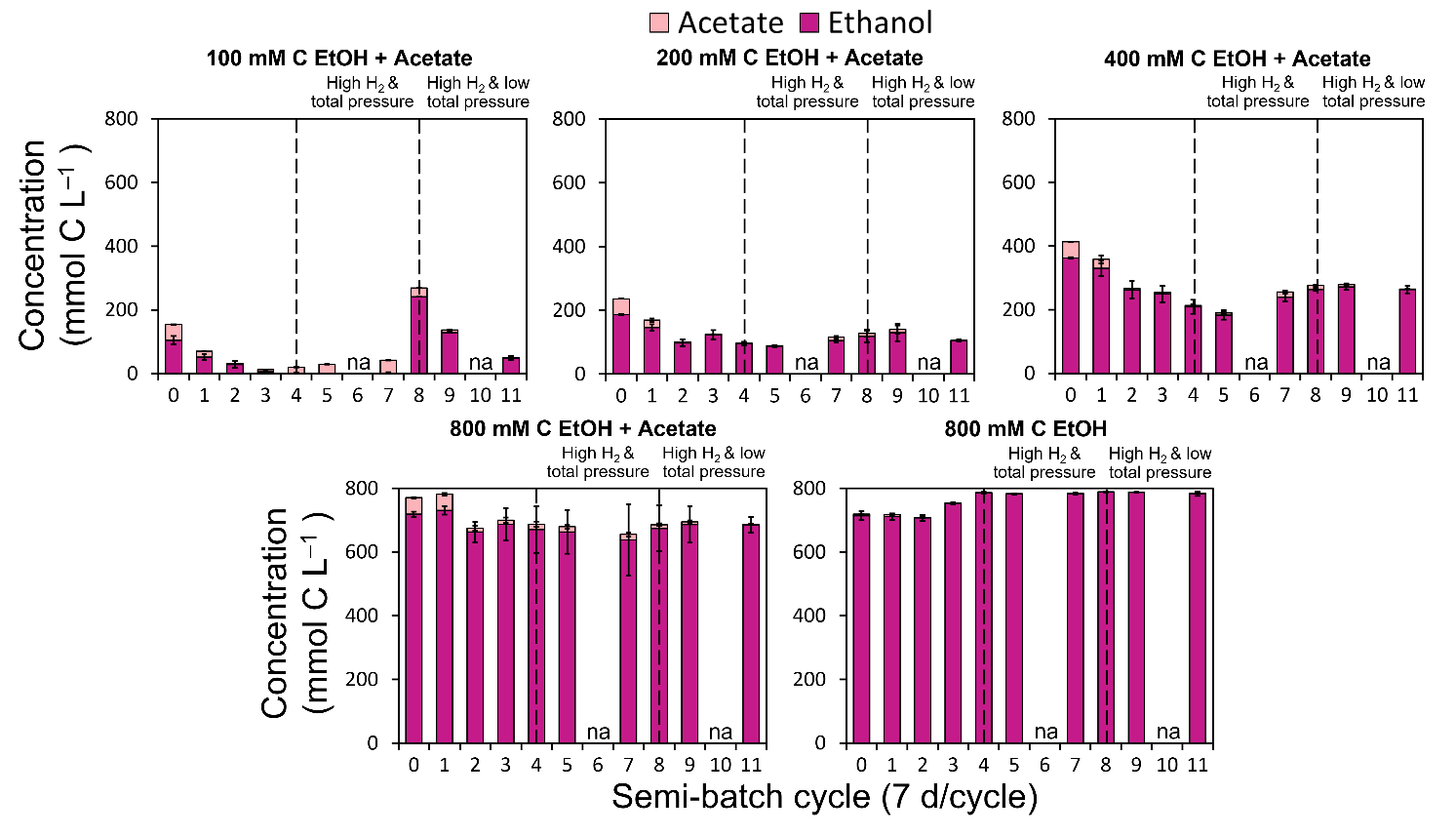


**Supplementary Figure S1.** Concentrations of acetate and ethanol in semi-batch bioreactors at time 0 and at the end of each cycle. The data are averages with standard deviations of triplicate bioreactors. na = not analyzed.


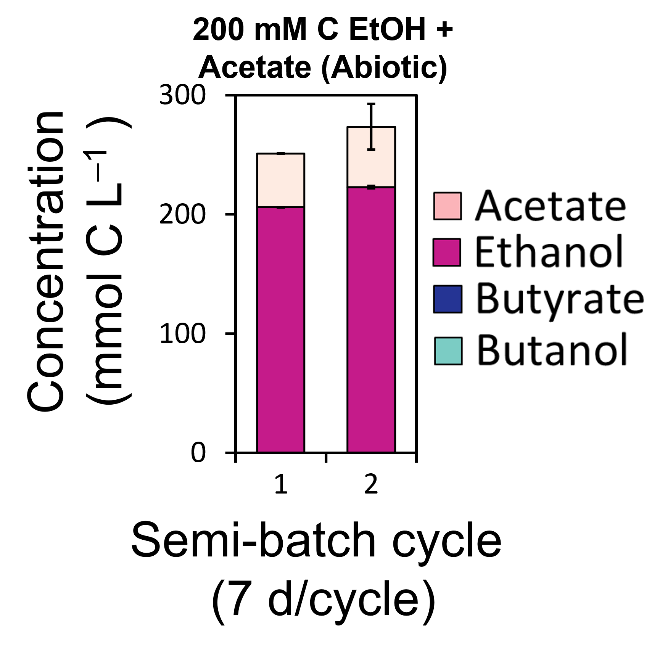


**Supplementary Figure S2.** Concentrations of chain elongation substrates and products in reactors with 200 mM C ethanol and 50 mM C acetate without an inoculum. These reactors were operated for 2 semi-batch cycles. The data are averages of with standard deviation of triplicate bioreactors.


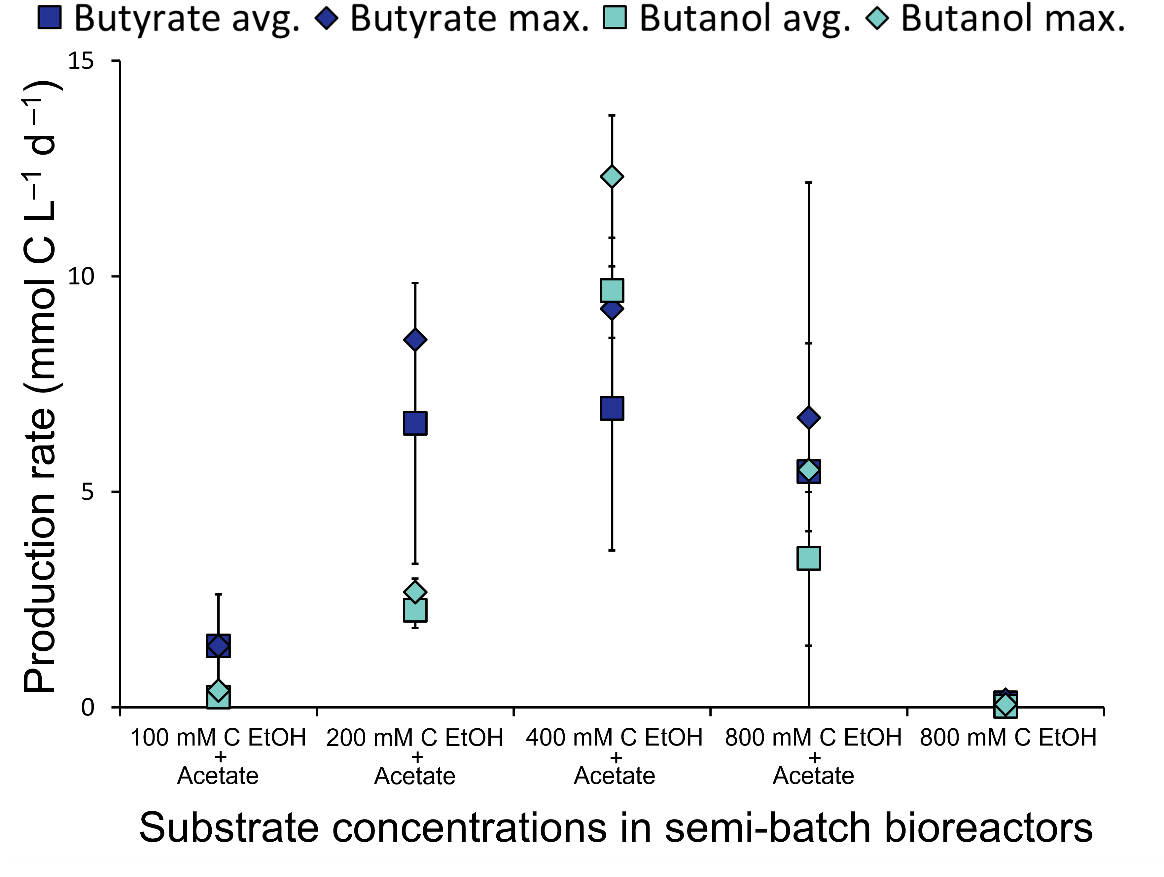


**Supplementary Figure S3.** Production rates of butyrate and butanol during “High H_2_ & total pressure” phase of the experiments. The average rates are the average with standard deviation of rates for semi-batch cycles 4, 5, and 7. The maximum rates show the average with standard deviation of the highest observed rate during an individual semi-batch cycle during the “High H_2_ & total pressure” phase of the experiment.


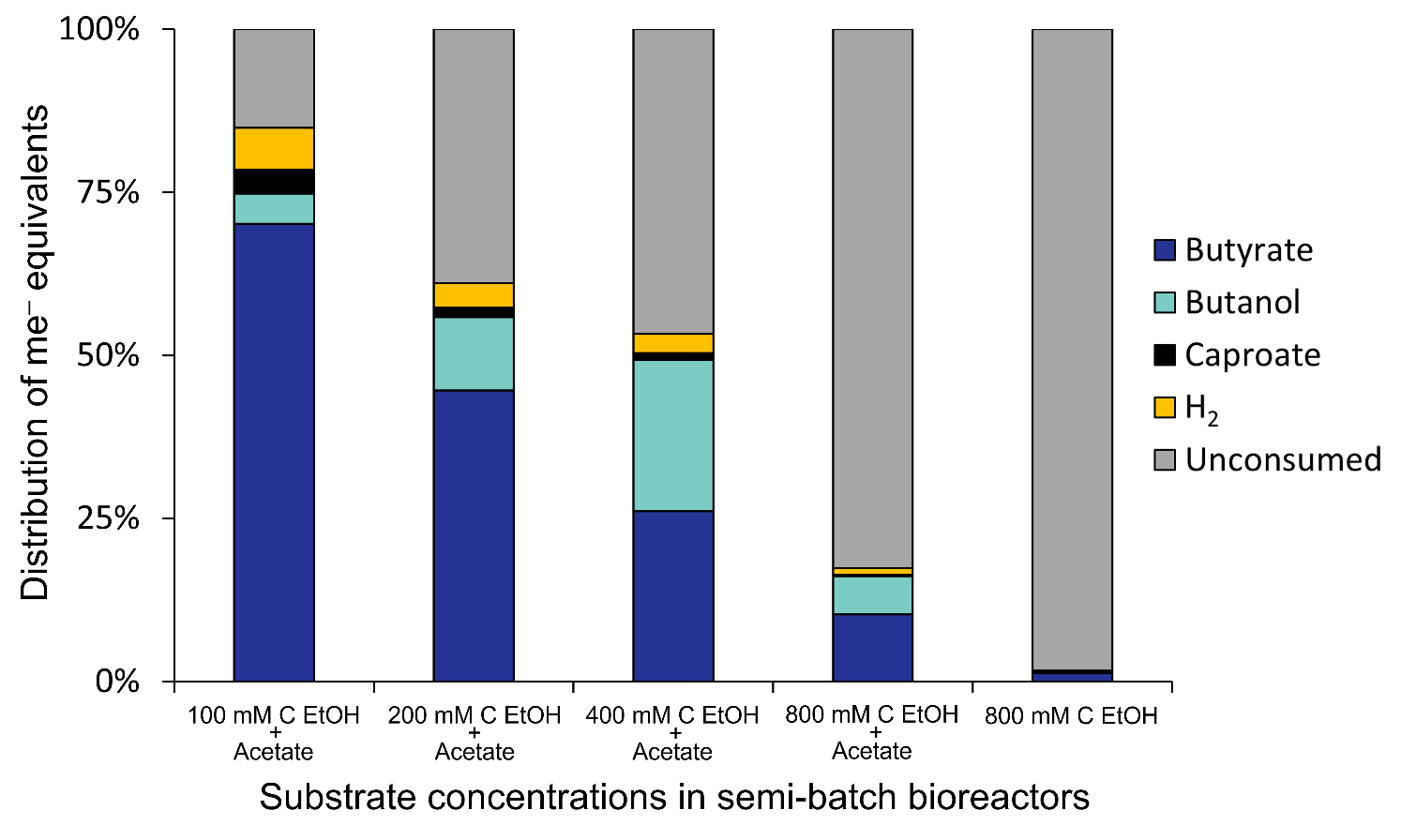


**Supplementary Figure S4**. Distribution of millielectron equivalents (me− equiv.) from acetate and ethanol (substrates) to end products at semi-batch cycle 4 (end of experiment) in bioreactors. The data are averages of triplicate bioreactors.


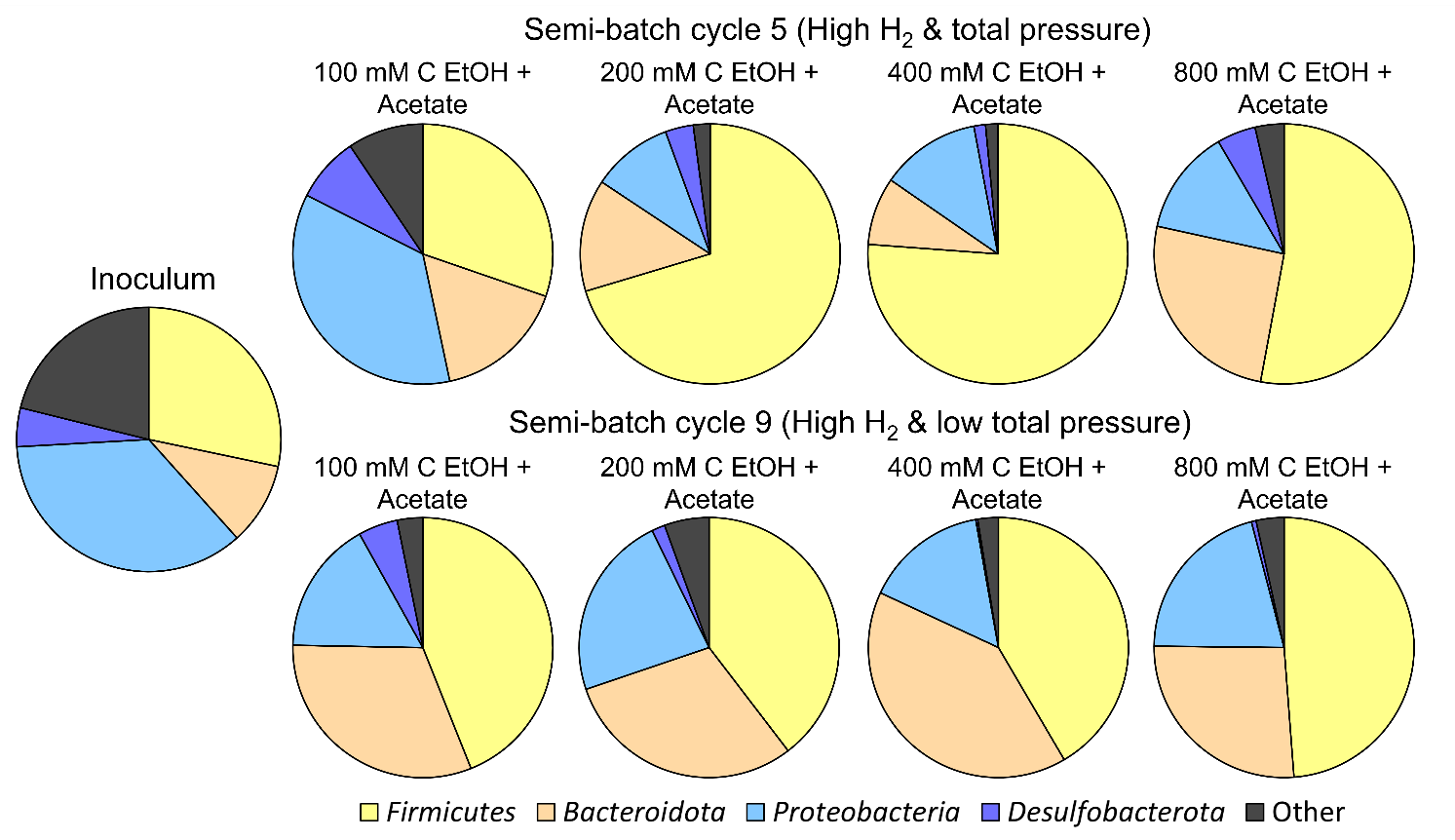


**Supplementary Figure S5.** Microbial community composition (at the phylum level) of the inoculum and in the bioreactors at the end of semi-batch cycles 5 and 9. The partial pressure in the bioreactors was decreased at the end of semi-batch cycle 7. The data are average sequences from triplicate bioreactors.


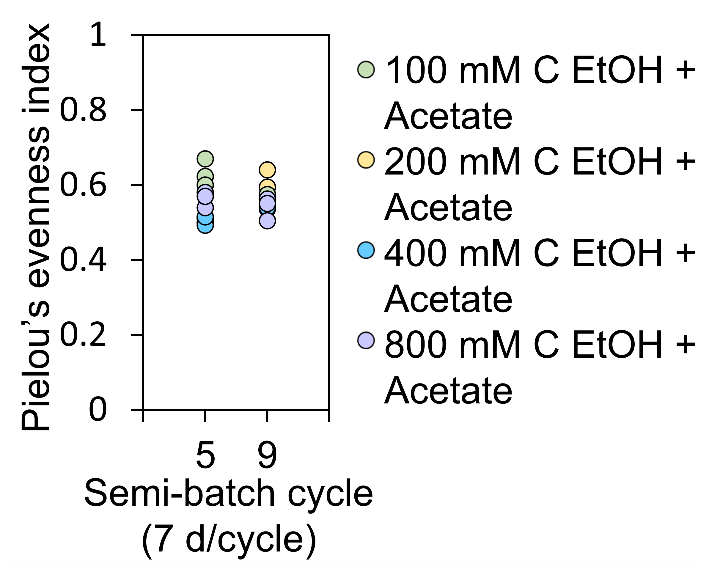


**Supplementary Figure S6.** Pielou’s evenness index values for semi-batch bioreactor samples taken during the “High H_2_ & total pressure” phase (cycle 5) and “High H_2_ & low total pressure” phase (cycle 9).
